# Early Post-Rewarming Fever Is Associated with Favorable 6-Month Neurologic Outcomes in Patients with Out-Of-Hospital Cardiac Arrest: A Multicenter Registry Study

**DOI:** 10.3390/jcm9092927

**Published:** 2020-09-10

**Authors:** Hyoung Youn Lee, Dong Hun Lee, Byung Kook Lee, Kyung Woon Jeung, Yong Hun Jung, Seung Phil Choi, Jung Soo Park, Jae Hoon Lee, Kap Su Han, Yong Il Min

**Affiliations:** 1 Department of Emergency Medicine, Chonnam National University Medical School, 160 Baekseo-ro, Dong-gu, Gwangju 61469, Korea; apostle09@naver.com (H.Y.L.); ggodhkekf@hanmail.net (D.H.L.); neoneti@hanmail.net (K.W.J.); xnxn77@hanmail.net (Y.H.J.); minyi46@hanmail.net (Y.I.M.); 2 Department of Emergency Medicine, Eunpyeong St. Mary’s Hospital, College of Medicine, The Catholic University of Korea, Seoul 03312, Korea; emvic98@catholic.ac.kr; 3 Department of Emergency Medicine, College of Medicine, Chungnam National University School of Medicine, 282 Mokdong-ro, Jung-gu, Daejeon 35015, Korea; Chopin-park@hanmail.net; 4 Department of Emergency Medicine, Dong-A University College of Medicine, 26 Daesingongwon-ro, Seo-Gu, Busan 49201, Korea; leetoloc@naver.com; 5 Department of Emergency Medicine, College of Medicine, Korea University, 73 Goryeodae-ro, Seongbuk-gu, Seoul 02841, Korea; hanks96@hanmail.net

**Keywords:** out-of-hospital cardiac arrest, targeted temperature management, prognosis, fever

## Abstract

We investigated the association between post-rewarming fever (PRF) and 6-month neurologic outcomes in cardiac arrest survivors. This was a multicenter study based on a registry of comatose adult (≥18 years) out-of-hospital cardiac arrest (OHCA) survivors who underwent targeted temperature management between October 2015 to December 2018. PRF was defined as peak temperature ≥ 38.0 °C within 72 h after completion of rewarming, and PRF timing was categorized as within 24, 24–48, and 48–72 h epochs. The primary outcome was neurologic outcomes at six months after cardiac arrest. Unfavorable neurologic outcome was defined as cerebral performance categories three to five. A total of 1031 patients were included, and 642 (62.3%) had unfavorable neurologic outcomes. PRF developed in 389 (37.7%) patients in 72 h after rewarming: within 24 h in 150 (38.6%), in 24–48 h in 155 (39.8%), and in 48–72 h in 84 (21.6%). PRF was associated with improved neurologic outcomes (odds ratio (OR), 0.633; 95% confidence interval (CI), 0.416–0.963). PRF within 24 h (OR, 0.355; 95% CI, 0.191–0.659), but not in 24–48 h or 48–72 h, was associated with unfavorable neurologic outcomes. Early PRF within 24 h after rewarming was associated with favorable neurologic outcomes.

## 1. Introduction

Fever or thermogenesis to maintain body temperature is a physiological reaction and important vital sign that is controlled by neuro-hormonal mechanisms to protect the body [1]. However, fever or elevated temperature is frequently observed after return of spontaneous circulation (ROSC) in cardiac arrest survivors and is associated with worse neurologic outcomes [2,3,4,5]. Hence, two landmark clinical trials proved that therapeutic hypothermia (TH) improves neurologic outcomes of comatose out-of-hospital cardiac arrest survivors (OHCA) [6,7]. Targeted temperature management (TTM) has been recommended as a standard treatment and is being practiced as a universal treatment based on accumulated evidence of favorable effects through additional high-quality clinical studies in various patients who experienced a cardiac arrest [8,9,10].

After TTM became a ubiquitously implemented treatment, it has been also discovered that fever develops even after the completion of TTM [11,12,13,14,15,16,17,18,19]. Although the impact of post-rewarming fever (PRF) that develops after the completion of TTM is inconclusive, experts recommend controlling the PRF [10,11,12,13,14,15,16,17,18,19]. PRF above 38.5 °C [11,12] or marked hyperthermia over 38.7 °C [13] was associated with worse outcomes, and guideline recommended that it is reasonable to actively prevent fever in comatose patients after TTM [10]. However, several studies failed to show the association between PRF and prognosis [14,15,16,17,18], whereas one observational study found that PRF within 48 h after TTM was associated with favorable neurologic outcomes [19]. Previous studies have analyzed mixed cohorts of OHCA and in-hospital cardiac arrest (IHCA) [13], included single-center populations [11,14,15,16,17], or used short-term clinical outcomes [12,13,14,15,16,17,18,19].

## 2. Experimental Section

### 2.1. Study Design and Population

The Korean Hypothermia Network established a prospective multicenter registry (KORHN-PRO; NCT02827422) of adults (≥18 years) OHCA who underwent TTM from 20 participating hospitals in South Korea between October 2015 and December 2018. This study was approved by the Institutional Ethics Committee at all participating hospitals as well as the Chonnam National University Institutional Review Board (CNUH-2015-164). A written informed consent was obtained from all patients in accordance with national requirements and the principle of the Declaration of Helsinki [20]. The registry excluded cases of pre-arrest Glasgow–Pittsburgh cerebral performance category (CPC) 3 or 4 [21]; temperature on admission < 30 °C; known disease resulting in unlikely survival at 180 days; and cardiac arrest caused by trauma, stroke, or intracranial bleeding. Independent researchers who were blinded to patient data assessed the 6-month neurologic outcomes using CPC and modified Rankin scores of either the patients or proxies. 

We included adult OHCA survivors who underwent TTM with a target temperature of 32–36 °C for 24 h and excluded patients (1) whose TTM was premature interrupted, (2) who were discharged or died within 24 h after achieved rewarming, (3) who had no data on the peak temperature during 72 h after completion of TTM, (4) who had a maintenance period other than 24 h, (5) who had a target temperature other than 32–36 °C and (6) who had a lack of data on 6-month neurologic outcomes.

### 2.2. Targeted Temperature Management

For TTM, participating hospitals cooled patients to a target temperature of 32–36 °C and recorded patient characteristics such as severity of brain injury, bleeding, or hemodynamic instability. All possible cooling methods were adopted with various cooling devices. Cooling was initiated as early as possible, and predetermined target duration was maintained after the achievement of target temperature. The temperatures were measured through core temperature sensor (98%) that was located in esophagus (80.6%), rectum (12.8%), and bladder (5.4%). Small proportion of patients (2%) were monitored the temperature through temperature sensor that was located axilla and tympanic membrane. Medication for analgosedation and neuromuscular blocking agents for control of shivering were given during TTM. Patients were rewarmed after the completion of maintenance duration at the rate of 0.2–0.5 °C/h. Body temperature was monitored after the completion of TTM to avoid the development of fever, and fever was actively controlled with anti-pyretic drugs or use of temperature management device as post rewarming temperature management (PRTM) depending on the local protocol to maintain normothermia at least 72 h after the completion of TTM. PRTM was implemented using temperature management device by leaving the cooling device in place after completion of rewarming. 

### 2.3. Data Collection

We extracted the following data from the KORHN-PRO registry: age; sex; comorbidities; etiology of cardiac arrest; witnessed arrest; bystander cardiopulmonary resuscitation (CPR); downtime; first monitored rhythm; initial arterial blood gas and lactate data after ROSC; maximal sequential organ failure assessment (SOFA) score within the first 2 days after ROSC [22]; presence of pre-TTM shock; pre-TTM temperature; time from ROSC to initiation of TTM; PRTM after the completion of rewarming; each peak temperature within 24 h, 24–48 h, 48–72 h and within 72 h after the completion of rewarming; seizure confirmed clinically or monitored using electroencephalography; infection; peak C-reactive protein (CRP) value within 1 week after ROSC; mortality and neurological status at hospital discharge and neurologic status at 6 months after cardiac arrest. PRF was defined as having a body temperature ≥ 38 °C within 72 h after rewarming [14]. Infection was defined in cases that needed antibiotic administration, including systemic inflammatory response syndrome, sepsis, pneumonia and other serious infections [23]. The primary outcome was unfavorable neurologic outcome, which was assessed using CPC at 6 months after the cardiac arrest and were defined as favorable (CPC 1 or 2) or unfavorable (CPC 3–5). The secondary outcome were the mortality and unfavorable neurologic outcome at hospital discharge.

### 2.4. Statistical Analysis

Continuous variables are reported as median values with interquartile ranges because all continuous variables had a non-normal distribution. Categorical variables are presented as frequency with percentages. Comparisons of continuous variables between independent groups were performed using the Mann–Whitney *U* test because of its non-normal distribution, and comparisons of categorical variables were performed using χ^2^ or Fisher’s exact tests, as indicated. A logistic regression analysis was conducted to identify independent risk factors for the development of PRF and neurologic outcomes after adjusting for relevant covariates. All variables with a *p*-value < 0.2 in the univariate analyses were included in the multivariate logistic regression model. Multicollinearity was assessed and variables with variance inflation factor >5 were excluded. Backward selection was used to reach the final model. To determine the contribution of peak temperature within 72 h as continuous variables, PRF and PRF timing (within 24, 24–48, and 48–72 h) as categorical variables on neurologic outcomes at hospital discharge or 6 months after cardiac arrest, we constructed another multivariate logistic regression model that had age, comorbidities, etiology of cardiac arrest, downtime, first monitored rhythm, witnessed arrest, bystander CPR, initial arterial blood gas data, peak CRP value, SOFA score, time from ROSC to initiation of TTM, pre-TTM temperature data, pre-TTM shock, seizure, and infection as covariates. Additionally, to determine the contribution of PRF and PRF timing (within 24, 24–48, and 48–72 h) on mortality at hospital discharge, we established another multivariate logistic regression model that had body mass index, etiology of cardiac arrest, downtime, first monitored rhythm, initial arterial blood gas data, peak CRP value, SOFA score, pre-TTM temperature data, and pre-TTM shock and seizure as covariates. Goodness of fit of the final model was evaluated using the Hosmer–Lemeshow test. We report logistic regression analysis results as odds ratio (OR) with 95% confidence interval (CI). Data were analyzed using SPSS version 18 software for windows (IBM Inc., Chicago, IL, USA). A two-sided significance level of 0.05 was used to indicate statistical significance.

## 3. Results

### 3.1. Study Population

**Figure 1:**
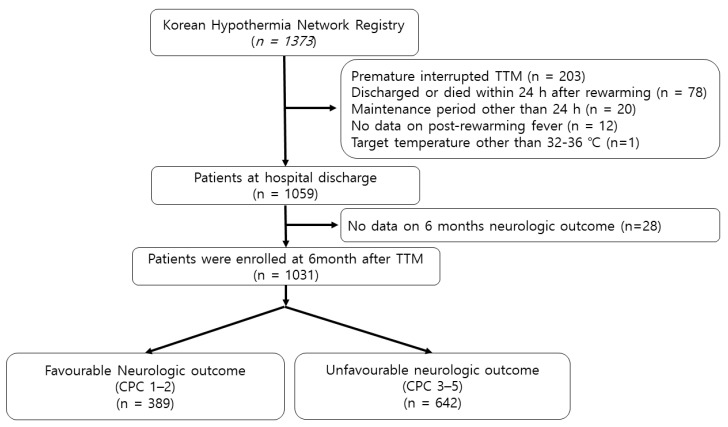
Flow diagram of the study. TTM, targeted temperature management; CPC, cerebral performance category scale.

A total of 1373 OHCA survivors who underwent TTM were registered in the KORHN-PRO registry. Of those, 203 patients were prematurely withdrawn from TTM, 78 died or were discharged within 24 h after TTM, 20 had a maintenance period over 24 h, 1 had target temperature other than 32–36 °C, 12 had no data for peak temperature during the 72 h after TTM, and 28 had no available data on neurologic outcomes at 6 months after the cardiac arrest (Figure 1).

Ultimately, 1059 patients at hospital discharge, and 1031 patients at 6 months after cardiac arrest were included in this study.

Table 1 shows baseline characteristics of the included patients. Unfavorable neurologic outcomes were observed in 642 (62.3%) patients. Patients with unfavorable neurologic outcomes were older and more female. In pre-existing illnesses, hypertension, diabetes mellitus, renal disease, pulmonary disease, and liver cirrhosis were significantly higher in patients with unfavorable outcomes, and these patients were more likely to have a non-cardiac etiology, unwitnessed arrest, no bystander CPR, and non-shockable rhythm. The duration of downtime was also significantly longer in these patients. After the ROSC, SOFA scores; incidence of pre-TTM shock; peak CRP value and complication rates of seizures, infections were higher in patients with unfavorable outcomes; these patients had significantly lower pH and HCO_3_ after ROSC and higher PaCO_2_ (Table 1). Peak temperature within 72 h after completion of TTM were lower in patients with unfavorable outcomes.

### 3.2. Factors Associated with Development of Post-Rewarming Fever

Table 2 shows characteristics stratified by PRF. PRF developed in 389 (37.7%) patients within 72 h after rewarming: in 24 h, 150 (38.6%) patients; in 24–48 h, 155 (39.8%) patients; and in 48–72 h, 84 (21.6%) patients. Patients with PRF were more male and younger than those without PRF (Table 1). In patients with PRF, the incidence of angina was significantly higher, whereas the incidence of renal and pulmonary disease was lower. Patients with PRF were more likely to have cardiac etiology, shockable rhythm, shorter downtime, lower SOFA scores, longer time from ROSC to initiation TTM, higher pre-TTM temperature and a lower rate of PRTM. The incidence of pre-TTM shock was also lower in these patients. Moreover, these patients had significantly higher pH and lower PaCO_2_. 

In a multivariate analysis to assess risk factors for PRF, younger age (OR, 0.988; 95% CI, 0.978–0.998), angina (OR, 2.336; 95% CI, 1.265–4.315), cardiac etiology (OR, 1.462; 95% CI, 1.067–2.004), lower SOFA score (OR, 0.878; 95% CI, 0.834–0.924), longer time from ROSC to initiation TTM (OR, 1.462; 95% CI, 1.067–2.004), higher pre-TTM temperature (OR, 1.214; 95% CI, 1.093–1.348), and PRTM (OR, 0.634; 95% CI, 0.470–0.856) were independently associated with PRF (Table 3).

### 3.3. Association between Post-Rewarming Fever and Mortality at Hospital Discharge

Table S1 shows baseline characteristics of the included patients according to the mortality at hospital discharge. Mortality rate at hospital discharge was 33.3% (353/1059). Non-survivor were older and high incidence of pre-existing diabetes mellitus and pulmonary disease. These patients were more likely to have a non-cardiac etiology, unwitnessed arrest, no bystander CPR, and non-shockable rhythm. The duration of downtime was also significantly longer in non survivors. After the ROSC, SOFA scores, incidence of pre-TTM shock, peak CRP value were higher and pre-TTM temperature were lower in non survivors. These patients had significantly lower pH and HCO_3_ after ROSC and higher PaCO_2_.

Table S2 shows result of multivariate analyses of mortality at hospital discharge. Body mass index (OR, 1.044; 95% CI, 1.002–1.088), cardiac etiology (OR, 0.473; 95% CI, 0.321–0.697), downtime (OR, 1.025; 95% CI, 1.015–1.036), shockable rhythm (OR, 0.535; 95% CI, 0.334– 0.857), SOFA score (OR, 1.114; 95% CI, 1.041–1.191), pH (OR, 0.221; 95% CI, 0.085–0.569), peak CRP (OR, 1.017; 95% CI, 1.006–1.028), pre-TTM temperature (OR, 0.816; 95% CI, 0.728–0.916), pre-TTM shock (OR, 1.671; 95% CI, 1.162–2.403), and seizure (OR, 0.682; 95% CI, 0.467–0.998) were independently associated with unfavorable neurologic outcomes.

PRF was not associated with mortality (OR, 0.732; 95% CI, 0.509–1.052). However, in the subgroup analysis, PRF within 24 h after rewarming compared to no PRF was independently associated with mortality, whereas PRF in 24–48 h or 48–72 h was not associated with mortality at hospital discharge (Table S2).

### 3.4. Association between Post-Rewarming Fever, and Timing of Post-rewarming Fever and Neurologic Outcomes at Hospital Discharge

Table S3 shows baseline characteristics of the included patients at hospital discharge. Unfavorable neurologic outcomes were observed in 667 (63.0%) patients at hospital discharge. Patients with unfavorable neurologic outcomes were older and more female. In pre-existing illnesses, hypertension, diabetes mellitus, angina, renal disease, pulmonary disease, and liver cirrhosis were significantly higher in patients with unfavorable outcomes, and these patients were more likely to have a non-cardiac etiology, unwitnessed arrest, no bystander CPR, and non-shockable rhythm. The duration of downtime was also significantly longer in these patients. After the ROSC, SOFA scores; incidence of pre-TTM shock; peak CRP value and complication rates of seizures, infections were higher in patients with unfavorable outcomes; these patients had significantly lower pH and HCO_3_ after ROSC and higher PaCO_2_ (Table 1). Peak temperature within 72 h after completion of TTM were lower in patient with unfavorable outcomes.

Table S4 shows results of multivariate analyses of unfavorable neurologic outcomes. Age (OR, 1.031; 95% CI, 1.016–1.047), angina (OR, 0.416; 95% CI, 0.187–0.923), cardiac etiology (OR, 0.274; 95% CI, 0.159–0.471), downtime (OR, 1.055; 95% CI, 1.039–1.070), shockable rhythm (OR, 0.237; 95% CI, 0.148– 0.381), SOFA score (OR, 1.089; 95% CI, 1.008–1.176), pH (OR, 0.107; 95% CI, 0.034–0.334), peak CRP (OR, 1.023; 95% CI, 1.009–1.038), pre-TTM temperature (OR, 0.737; 95% CI, 0.633–0.857), seizure (OR, 5.984; 95% CI, 3.655–9.797), and infection (OR, 1.573; 95% CI, 1.043–2.372) were independently associated with unfavorable neurologic outcomes.

The final multivariate logistic model demonstrated lower peak temperature within 72 h after rewarming was independently associated with unfavorable neurologic outcome (OR, 0.641; 95% CI, 0.471–0.872). PRF was independently associated with unfavorable neurologic outcomes (OR, 0.623; 95% CI, 0.410–0.945). In the subgroup analysis, PRF within 24 h after rewarming compared to no PRF was independently associated with improved neurologic outcomes, whereas PRF in 24–48 h or 48–72 h was not associated with neurologic outcomes at hospital discharge (Table S4).

### 3.5. Association of Post-Rewarming Fever, and Timing of Post-Rewarming Fever and Neurologic Outcomes

**Figure 2:**
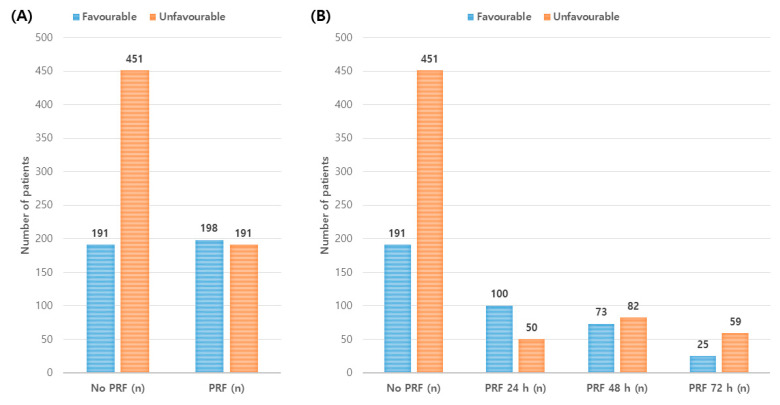
Association of neurological outcomes at 6 months with post-rewarming fever and timing of post-rewarming fever. (**A**) Patients with post-rewarming fever (PRF) had higher favorable neurologic outcomes than those without PRF (*p* < 0.001). (**B**) Neurological outcomes were different between PRF onset timings (*p* < 0.001). Patients who developed PRF within the 24 h, 24–48 h, and 48–72 h had 66.7%, 47.1%, and 29.8% favorable neurologic outcomes, respectively.

Patients with PRF more often had favorable neurologic outcomes than those without PRF (*p* < 0.001), and neurologic outcomes were different among patients with different PRF timing (Figure 2). Table 4 shows results of multivariate analyses of unfavorable neurologic outcomes. Age (OR, 1.032; 95% CI, 1.017–1.047), cardiac etiology (OR, 0.246; 95% CI, 0.146–0.413), downtime (OR, 1.053; 95% CI, 1.038–1.069), shockable rhythm (OR, 0.218; 95% CI, 0.136– 0.349), SOFA score (OR, 1.109; 95% CI, 1.027–1.197), pH (OR, 0.131; 95% CI, 0.042–0.410), peak CRP (OR, 1.025; 95% CI, 1.010–1.039), pre-TTM temperature (OR, 0.727; 95% CI, 0.626–0.846), seizure (OR, 5.262; 95% CI, 3.243–8.538), and infection (OR, 1.691; 95% CI, 1.122–2.550) were independently associated with unfavorable neurologic outcomes.

The final multivariate logistic model demonstrated PRF and peak temperature within 72 h after TTM was independently associated with unfavorable neurologic outcomes (OR, 0.633; 95% CI, 0.416–0.963 and OR, 0.660; 95% CI, 0.485–0.899, respectively). Moreover, PRF within 24 h after rewarming compared to no PRF was independently associated with improved neurologic outcomes, whereas PRF in 24–48 h or 48–72 h was not associated with neurologic outcomes (Table 4).

## 4. Discussion

In this multicenter registry-based study, PRF and increased peak temperature within 72 h after rewarming were significantly associated with favorable neurologic outcomes at hospital discharge and 6 months after cardiac arrest. In the subgroup analyses, PRF within 24 h after the completion of TTM was significantly associated with neurologic outcomes rather than PRF in 24–48 h or 48–72 h. However, there was no significant association between PRF and survival discharge. In the subgroup analyses, significant association was showed only between PRF within 24 h after rewarming and mortality at hospital discharge.

In the era of TTM, there are wide variabilities in the association between the development of fever after TTM and clinical outcomes [11,12,13,14,15,16,17,18,19]. Some studies demonstrated that there were significant association between worse neurologic outcomes and PRF [11,12], or marked hyperthermia [13,24] but, others have failed to show these results [14,15,16,17,18]. A multicenter observational study using the Penn Alliance for Therapeutic Hypothermia Registry reported that PRF was not associated with survival and neurologic outcomes in OHCA survivors, even though, the higher maximum temperature (≥ 38.8 °C) was associated with worse outcomes in OHCA patients [24]. Contrary to these prior studies, one retrospective observational study found that elevated temperature within 48 h after TTM was associated with favorable neurologic outcomes regardless of the control of PRF [19]. In this study, the first (35.3–36.2 °C) or second (36.2–36.5 °C) quartile was independently associated with unfavorable neurologic outcomes compared with the highest quartile (36.8–37.6°C) [19]. Therefore, mild pyrexia following TTM may be an indicator of favorable outcomes in cardiac arrest survivors. In line with the findings of a previous study, PRF within 72 h after TTM was significantly associated with favorable neurologic outcomes in this study. Fever or thermogenesis is physiological and may represent viability of the neurohormonal pathways [1]. Although fever reaction or thermogenesis itself may not guarantee intact neurological integrity of the whole brain including the neocortex that is vulnerable to ischemia [25], elevated temperatures in patients experiencing cardiac arrests may represent the viability of deep brain structures such as the preoptic and anterior hypothalamus, which is believed to play a principal role in thermoregulation [26]. However, whether PRF is a consequence of damage or cause of neurologic outcomes remains unknown and requires further investigations. 

It is likely that earlier fever after ROSC implies worse neurologic outcomes in the pre-TTM era [2,3,4,5]. Laboratory studies have demonstrated that induced hyperthermia at 24 h after ROSC worsened neurologic outcomes rather than induced hyperthermia at 48 h after ROSC [27]. However, it is still unknown whether earlier PRF is unfavorable in cardiac arrest survivors in the TTM era. Contrary to previous research, we found that PRF within 24 h after TTM was a robust indicator of favorable neurologic outcomes. PRF might be a result of a continuous effort of thermogenesis that was suppressed during TTM. An observational study showed that the TH group had delayed fever compared to the non-TH group (15 h vs. 36 h) [18]. Another study that investigated the effects of post-rewarming temperature management (PRTM) that continued additional 24 h to maintain the core temperature as 36 °C with or without PRF demonstrated that PRTM was associated with delayed PRF not with the prevention of PRF [19]. In the present study, PRTM showed the significant association with lower PRF, but not with the neurologic outcome. The association between PRF and favorable neurologic outcome in the present study might partially explain that PRTM fail to improve neurologic outcome. However, it is not conclusive, since PRTM was different between hospitals according to their protocol regarding duration and device. The early recovery of fever reaction may indicate early resurrection of the deep brain as part of the limbic system and thereby, it may be an indicator of brain recovery. 

One study investigated heat generation during the maintenance period of TTM and demonstrated that greater heat generation was associated with better neurologic outcomes [28]. Higher temperature before the initiation of TTM or prolonged induction period that presumed to resist against cooling was also associated with favorable neurologic outcomes [17,29]. Similarly, higher pre-TTM temperature was significantly associated with favorable neurologic outcomes in this study. However, several previous studies showed that fever early after resuscitation was detrimental in neurologic prognosis [2,3,4,5,11,12,18,24]. It is presumed that neuronal vulnerability early after resuscitation may contribute to intolerance to fever [18]. Early fever can be a double-edged sword: one side as a positive sign of favorable neurologic outcomes, and the other side as a detrimental risk factor for neuronal damage. Blunting the detrimental side of fever by suppression of fever might be a core role of TTM for resuscitation. In the present study, independent risk factors of PRF development were younger age, angina history, cardiac etiology, lower SOFA score, longer time from ROSC to initiation of TTM, higher pre-TTM temperature and PRTM. Active PRF management with modulation based on these risk factors may be helpful for the treatment of patients who experience a cardiac arrest.

There are several limitations of this study. First, PRF was based on the peak temperature for each day within three days after rewarming; therefore, we were not able to analyze data regarding fever interval and duration. We also had missing data on peak temperature of 24–48 h and 48–72 h in 17 (1.7%) patients and 64 (6.2%) patients, respectively. Second, there were wide variations in the target temperatures of TTM, from 32 °C to 36 °C. Although, there was no association between target temperature and PRF, it may imply a difference in patient characteristics or depth of TTM. However, a target temperature of 32–36 °C is the standard protocol in guidelines [10]. Therefore, our results are generalizable. Third, the site of temperature measurement was not standardized, although majority of temperatures were measured with a core temperature sensor (98.0%) that was located in different core body region. Moreover, we were not able to identify the temperature measurement interval after completion of rewarming, the result might be biased. However, PRF was measured after the active TTM procedures which can cause temperature difference between core body regions. One study about intensive care patients without any active temperature management procedures showed that the mean differences of core temperature were small [30]. Forth, in South Korea, decisions regarding the termination of life support were not allowed, but the ‘Well-Dying Law’ for terminally ill patients has enacted from February 2018 which allows to stop the additional active treatment. Among the included patient in this study, 40 (3.9%) patients accepted the limitation of additional active treatment whereas almost all the previous patients did not receive this. Therefore, the prognostic results might have confounded. Fifth, favorable patients who regain consciousness within a short period of time after TTM did not need to receive the PRTM thoroughly, so PRTM might be a confounder of the results. Hence, this characteristic of PRTM might affect the no association between PRTM and neurologic outcome at discharge or six months. Additionally, protocols of PRF management were also not standardized including inconsistency of TTM devices and PRTM depth or duration; therefore, we followed general guidelines for recommendations on how to avoid fevers [10].

## 5. Conclusions

In this multicenter registry-based study, PRF within 72 h following TTM was associated with favorable neurologic outcomes at hospital discharge and 6 months after cardiac arrest in OHCA patients treated with TTM. However, PRF within 72 h following TTM was not associated with mortality at hospital discharge. The occurrence of PRF within 24 h, but not in 24–48 h or 48–72 h, after rewarming was associated with favorable neurologic outcomes and decreased mortality.

## Figures and Tables

**Table 1:** Demographics and characteristics of the included patients according to neurologic outcomes at 6 months after hospital discharge.

Variables	Total (*n* = 1031)	Favorable (*n* = 389)	Unfavorable (*n* = 642)	*p*-Value
Age, years, IQR	57 (46–68)	54 (44–62)	60 (48–71)	<0.001
Male sex, *n* (%)	737 (71.5)	303 (77.9)	434 (67.6)	<0.001
Body mass index, kg m^–2^, IQR	23.2 (20.9–25.7), 1023 *	23.2 (21.3–25.6), 387 *	23.2 (20.7–25.8), 636 *	0.721
Pre-existing illness				
Hypertension, *n* (%)	358 (34.7)	115 (29.6)	243 (37.9)	0.007
Diabetes mellitus, *n* (%)	232 (22.5)	61 (15.7)	171 (26.6)	<0.001
AMI, *n* (%)	65 (6.3)	27 (6.9)	38 (5.9)	0.597
Angina, *n* (%)	64 (6.2)	34 (8.7)	30 (4.7)	0.011
Congestive heart failure, *n* (%)	36 (3.5)	12 (3.1)	24 (3.7)	0.606
Arrhythmia, *n* (%)	50 (4.8)	21 (5.4)	29 (4.5)	0.551
Renal disease, *n* (%)	79 (7.7)	17 (4.4)	62 (9.7)	0.002
Pulmonary disease, *n* (%)	60 (5.8)	8 (2.1)	52 (8.1)	<0.001
TIA or ischemic stroke, *n* (%)	59 (5.7)	18 (4.6)	41 (6.4)	0.270
Liver cirrhosis, *n* (%)	12 (1.2)	1 (0.3)	11 (1.7)	0.037
Malignancy, *n* (%)	55 (5.3)	20 (5.1)	35 (5.5)	0.887
Cardiac etiology, *n* (%)	644 (62.5)	340 (87.4)	304 (47.4)	<0.001
Downtime, min, IQR	27 (16–40)	18 (12–27)	33 (22–46)	<0.001
Witness, *n* (%)	730 (70.8)	325 (83.5)	405 (63.1)	<0.001
Bystander CPR, *n* (%)	648 (62.9)	262 (67.4)	386 (60.1)	0.020
Shockable rhythm, *n* (%)	379 (36.8)	269 (71.0)	110 (17.1)	<0.001
SOFA score, IQR	11 (8–12), 954 *	9 (7–11), 369 *	11 (9–13), 585 *	<0.001
Serum lactate, mg dL^–1^, IQR	9.4 (5.8–12.5), 997 *	9.4 (5.5–12.4), 377 *	9.4 (6.0–12.6), 620 *	0.481
pH, IQR	7.11 (6.94–7.25), 997 *	7.22 (7.09–7.30), 371 *	7.04 (6.89–7.18), 626 *	<0.001
PaCO_2_, mmHg, IQR	47 (35.0–69.0), 997 *	39.0 (32.0–49.0), 371 *	56.0 (38.8–80.1), 626 *	<0.001
HCO_3_, mEq dL^–1^, IQR	15.3 (12.0–18.8), 995 *	15.8 (12.9–19.1), 371 *	15.0 (11.6–18.8), 624 *	0.028
Peak CRP, mg dL^–1^, IQR	15.8 (9.4–24.3), 994 *	12.9 (7.4–18.8), 371 *	18.1 (11.4–27.0), 623 *	<0.001
Time from ROSC to initiation of TTM, min	210.0 (132.0–301.0)	214.0 (146.0–299.0)	208.0 (122.8–304.3)	0.163
Pre-TTM Temperature (°C), IQR	36 (34.9–36.8), 1006 *	36.4 (35.8–37.0), 379 *	35.6 (34.5–36.4), 627 *	<0.001
Peak temperature, (°C), within 72 h, IQR	37.7 (37.2–38.2)	38.0 (37.6–38.3)	37.5 (37.0–38.0)	<0.001
Pre-TTM Shock, *n* (%)	474 (46.0)	132 (33.9)	342 (53.3)	<0.001
PRTM, *n* (%)	648 (62.9)	248 (63.8)	400 (62.3)	0.642
Target temperature, *n* (%)				0.812
32.0−34.0 °C	820 (79.5)	311 (79.9)	509 (79.3)	
34.1−36.0 °C	211 (20.5)	78 (20.1)	133 (20.7)	
Seizure, *n* (%)	279 (27.1)	51 (13.1)	228 (35.5)	<0.001
Infection, *n* (%)	563 (54.6)	190 (48.8)	373 (58.1)	0.004

Data are presented as the *n* (%) for categorical variables, unless otherwise indicated. IQR, interquartile range; AMI, acute myocardial infarction; TIA, transient ischemic attack; CPR, cardiopulmonary resuscitation; SOFA, sequential organ failure assessment; PaCO_2_, partial pressure of arterial carbon dioxide tension; HCO_3_, arterial bicarbonate; TTM, targeted temperature management; CRP, C-reactive protein; ROSC, return of spontaneous circulation; PRTM, post rewarming temperature management. * Included number for analyses.

**Table 2:** Demographics and characteristics of the included patients according to post-rewarming fever.

Variables	PRF (*n* = 389)	No PRF (*n* = 642)	*p*-Value
Age, years, IQR	56 (45–66)	58 (48–69)	0.007
Male sex, *n* (%)	302 (77.6)	435 (67.8)	0.001
Body mass index, kg m^–2^, IQR	23.6 (21.4–25.9), 386 *	23.1 (20.7–25.4), 637 *	0.066
Pre-existing illness			
Hypertension, *n* (%)	128 (32.9)	230 (35.8)	0.346
Diabetes mellitus, *n* (%)	77 (19.8)	155 (24.1)	0.107
AMI, *n* (%)	25 (6.4)	40 (6.2)	1.000
Angina, *n* (%)	32 (8.2)	32 (5.0)	0.045
Congestive heart failure, *n* (%)	12 (3.1)	24 (3.7)	0.606
Arrhythmia, *n* (%)	20 (5.1)	30 (4.7)	0.766
Renal disease, *n* (%)	20 (5.1)	59 (9.2)	0.021
Pulmonary disease, *n* (%)	14 (3.6)	46 (7.2)	0.019
TIA or ischemic stroke, *n* (%)	20 (5.1)	39 (6.1)	0.582
Liver cirrhosis, *n* (%)	2 (0.5)	10 (1.6)	0.229
Malignancy, *n* (%)	14 (3.6)	41 (6.4)	0.063
Cardiac etiology *n* (%)	270 (69.4)	374 (58.3)	<0.001
Downtime, min, IQR	27 (16–40)	30 (18–43)	<0.001
Witness *n* (%)	279 (71.7)	451 (70.2)	0.622
Bystander CPR *n* (%)	239 (61.4)	409 (63.7)	0.506
Shockable rhythm, *n* (%)	176 (46.4)	203 (31.6)	<0.001
SOFA score, IQR	10 (7–12), 364 *	11 (9–13), 590 *	<0.001
Serum lactate, mg dL^–1^, IQR	9.2 (5.5–12.5), 383^*^	9.5 (6.0–12.5), 614 *	0.161
pH, IQR	7.16 (7.00–7.26), 376 *	7.08 (6.92–7.24), 621 *	<0.001
PaCO_2_, mmHg, IQR	44.3 (34.3–63.0), 376 *	49.2 (35.9–73.0), 621 *	0.008
HCO_3_, mEq dL^–1^, IQR	15.3 (12.4–18.9), 375 *	15.3 (11.8–18.8), 620 *	0.580
Peak CRP, mg dL^–1^, IQR	15.8 (10.6–22.4), 369 *	15.7 (8.6–25.6), 625 *	0.933
Time from ROSC to initiation of TTM, min	224 (152.5–330.5)	201.0 (123.0–289.3)	<0.001
Pre-TTM Temperature, °C, IQR	36.2 (35.4–37.0), 380 *	35.8 (34.6–36.5), 626 *	<0.001
Pre-TTM Shock, *n* (%)	155 (39.8)	319 (49.7)	0.001
PRTM, *n* (%)	217 (55.8)	431 (67.1)	<0.001
Target temperature of TTM, *n* (%)			0.691
32.0–34.0 °C	312 (80.2)	508 (79.1)	
34.1–36.0 °C	77 (19.8)	134 (20.9)	
Seizure *n* (%)	113 (29.0)	166 (25.9)	0.278
Infection *n* (%)	228 (58.6)	335 (52.2)	0.046

Data are presented as the *n* (%) for categorical variables, unless otherwise indicated. PRF, post-rewarming fever; IQR, interquartile range; AMI, acute myocardial infarction; TIA, transient ischemic attack; CPR, cardiopulmonary resuscitation; SOFA, sequential organ failure assessment; PaCO_2_, partial pressure of arterial carbon dioxide tension; HCO_3_, arterial bicarbonate; TTM, targeted temperature management; CRP, C-reactive protein; ROSC, return of spontaneous circulation; PRTM, post rewarming temperature management. * Included number for analyses.

**Table 3:** Multivariate logistic regression analysis to assess independent risk factors for the development of post-rewarming fever.

Variables	AOR (95% CI)	*p*-Value
Age, years	0.988 (0.978–0.998)	0.015
Male sex	1.323 (0.950–1.844)	0.098
Body mass index, kg m^–2^	1.032 (0.996–1.071)	0.084
Angina	2.336 (1.265–4.315)	0.007
Diabetes mellitus	1.114 (0.755–1.643)	0.588
Renal disease	0.723 (0.380–1.375)	0.323
Pulmonary disease	0.726 (0.360–1.467)	0.373
Malignancy	0.569 (0.291–1.114)	0.100
Cardiac etiology	1.462 (1.067–2.004)	0.018
Downtime, min	0.993 (0.984–1.002)	0.119
Shockable rhythm	1.164 (0.792–1.712)	0.440
SOFA score	0.878 (0.834–0.924)	<0.001
Lactate, mg dL^–1^	0.993 (0.965–1.021)	0.610
pH	0.631 (0.273–1.458)	0.281
PaCO_2_, mmHg	0.999 (0.990–1.007)	0.804
Time from ROSC to initiation of TTM, min	1.002 (1.001–1.002)	<0.001
Pre-TTM shock	0.833 (0.602–1.152)	0.270
Pre-TTM temperature, °C	1.214 (1.093–1.348)	<0.001
PRTM	0.634 (0.470–0.856)	0.003
Infection	1.340 (0.997–1.802)	0.053

AOR, adjusted odds ratio; CI, confidence interval; SOFA, sequential organ failure assessment; PaCO_2_, partial pressure of arterial carbon dioxide tension; TTM, targeted temperature management; ROSC, return of spontaneous circulation; PRTM, post rewarming temperature management.

**Table 4:** Multivariate logistic regression analyses of post-rewarming fever and onset-time of post-rewarming fever for unfavorable neurologic outcome at 6 months after cardiac arrest.

Variables	AOR (95% CI)	AOR (95% CI)	AOR (95% CI)	AOR (95% CI)
Age, years	1.032 (1.017–1.047)	1.029 (1.014–1.044)	1.030 (1.015–1.045)	1.030 (1.015–1.045)
Male sex	0.723 (0.459–1.138)			
Hypertension	1.374 (0.850–2.221)			
Diabetes mellitus	1.320 (0.765–2.277)			
Angina	0.613 (0.278–1.354)			
Renal disease	0.818 (0.346–1.932)			
Pulmonary disease	0.734 (0.268–2.005)			
Liver cirrhosis	10.142 (0.731–140.716)			
Cardiac etiology	0.246 (0.146–0.413)	0.255 (0.152–0.430)	0.251 (0.149–0.423)	0.256 (0.152–0.434)
Downtime, min	1.053 (1.038–1.069)	1.051(1.035–1.066)	1.051 (1.036–1.067)	1.050 (1.035–1.065)
Witness	0.752 (0.454–1.246)			
Bystander CPR	1.305 (0.848–2.008)			
Shockable rhythm	0.218 (0.136–0.349)	0.235 (0.147–0.377)	0.228 (0.142–0.364)	0.219 (0.136–0.353)
SOFA score	1.109 (1.027–1.197)	1.109 (1.027–1.197)	1.107 (1.026–1.195)	1.102 (1.020–1.190)
pH	0.131 (0.042–0.410)	0.123 (0.039–0.386)	0.126 (0.040–0.396)	0.125 (0.039–0.393)
PaCO_2_, mmHg	1.006 (0.985–1.027)			
HCO_3_, mEq dl^–1^	1.036 (0.991–1.084)			
Peak CRP, mg dL^–1^, IQR	1.025 (1.010–1.039)	1.024 (1.010–1.039)	1.024 (1.009–1.038)	1.024 (1.009–1.038)
Time from ROSC to initiation of TTM, min	1.000 (0.999–1.002)			
Pre-TTM Temperature, °C	0.727 (0.626–0.846)	0.752 (0.646–0.876)	0.745 (0.640–0.867)	0.764 (0.656–0.890)
Pre-TTM Shock	1.150 (0.732–1.806)			
Seizure	5.262 (3.243–8.538)	5.308 (3.270–8.618)	5.320 (3.278–8.634)	5.443 (3.330–8.897)
Infection	1.691 (1.122–2.550)	1.763 (1.165–2.669)	1.690 (1.121–2.547)	1.614 (1.067–2.443)
Peak temperature, °C, within 72 h	NA	0.660 (0.485–0.899)	NA	NA
PRF	NA		0.633 (0.416–0.963)	NA
No PRF	NA		NA	Reference
PRF within 24 h	NA		NA	0.355 (0.191–0.659)
PRF 24–48 h	NA		NA	0.797 (0.458–1.386)
PRF 48–72 h	NA		NA	0.980 (0.463–2.074)

AOR, adjusted odds ratio; CI, confidence interval; IQR, interquartile range; CPR, cardiopulmonary resuscitation; SOFA, sequential organ failure assessment; PaCO_2_, partial pressure of arterial carbon dioxide tension; HCO_3_, arterial bicarbonate; TTM, targeted temperature management; PRF, post-rewarming fever; CRP, C-reactive protein; ROSC, return of spontaneous circulation; NA, not applicable.

## Supplementary Materials

The following are available online at https://www.mdpi.com/2077-0383/9/9/2927/s1, Table S1: Demographics and characteristics of the included patients according to mortality at hospital discharge, Table S2: Multivariate logistic regression analyses of post-rewarming fever and onset-time of post-rewarming fever for mortality at hospital discharge, Table S3: Demographics and characteristics of the included patients according to neurologic outcomes at hospital discharge, Table S4. Multivariate logistic regression analyses of post-rewarming fever and onset-time of post-rewarming fever for unfavorable neurologic outcome at hospital discharge.
