# Supplementary material for: Early Post-Rewarming Fever Is Associated with Favorable 6-Month Neurologic Outcomes in Patients with Out-Of-Hospital Cardiac Arrest: A Multicenter Registry Study
Source: J Clin Med. 2020 Sep 10;9(9):2927. doi: 10.3390/jcm9092927 (PMC7565524; doi:10.3390/jcm9092927)
Supplement: Supplementary file 1 [file jcm-09-02927-s001.pdf]

Journal of
Clinical Medicine

Supplementary materials

Table 1. Demographics and characteristics of the included patients according to mortality at hospital discharge.

	Total (n = 1059)	Survival (n = 706)	Death (n = 353)	p-Value
Age, years, IQR	57.0(46.0–68.0)	57.0(46.0–67.0)	59.0(47.0–71.0)	0.007
Male sex, n (%)	758 (71.6)	510(72.2)	248(70.3)	0.516
Body mass index, kg m^{-2}, IQR	23.2(20.9–25.7)	23.1(20.8–25.4), 742*	23.6(21.1–26.2), 349*	0.083
Pre-existing illness				
Hypertension, n (%)	367 (34.7)	236(33.4)	121(37.1)	0.245
Diabetes mellitus, n (%)	238 (22.5)	141(20.0)	97(27.5)	0.006
AMI, n (%)	65 (6.1)	45(6.4)	20(5.7)	0.686
Angina, n (%)	64 (6.0)	48(6.8)	16(4.5)	0.171
Congestive heart failure, n (%)	37 (3.5)	25(3.5)	12(3.4)	1.000
Arrhythmia, n (%)	52(4.9)	40(5.7)	12(3.4)	0.131
Renal disease, n (%)	80 (7.6)	46(6.5)	34(9.6)	0.084
Pulmonary disease, n (%)	66 (6.2)	35(5.0)	31(8.8)	0.021
TIA or ischemic stroke, n (%)	61(5.8)	41(5.8)	20(5.7)	1.000
Liver cirrhosis, n (%)	12 (1.1)	8(1.1)	4(1.1)	1.000
Malignancy, n (%)	56(5.3)	40(5.7)	16(4.5)	0.470
Cardiac etiology, n (%)	662(62.5)	497(70.4)	165(46.7)	<0.001
Downtime, min, IQR	27.0(16.0–40.0)	23.0(14.0–35.0)	36(24.5–49.0)	<0.001
Witness, n (%)	746(70.4)	530(75.1)	216(61.2)	<0.001
Bystander CPR, n (%)	664(62.7)	452(64.0)	212(60.1)	0.225
Shockable rhythm, n (%)	388(36.6)	328(46.5)	60(17.0)	<0.001
SOFA score, IQR	11.0(8.0–12.0)	10.0(7.0–12.0), 662 *	12.0(10.0–13.0), 315 *	<0.001
Serum lactate, mg dL^{-1}, IQR	9.4(5.8–12.5)	9.2(5.5–12.4), 686 *	9.7(6.5–13.0), 338 *	0.038
pH, IQR	7.11(6.94–7.25)	7.17(7.00–7.28), 680 *	7.02(6.87–7.15), 343 *	<0.001
$PaCO_2$, mmHg, IQR	47.0(35.0–69.0)	43.2(33.65–62.7), 680 *	56.7(41.0–81.2), 343 *	<0.001
HCO_3, mEq dL^{-1}, IQR	15.3(12.0–18.8)	15.7(12.7–19.1),678 *	14.8(11.1–18.7), 343 *	0.003
Peak CRP, mg dL^{-1}, IQR	15.8(9.45–12.45)	14.1(8.6–21.1), 681 *	20.5(12.33–29.48), 336 *	<0.001
Time from ROSC to initiation of TTM, min	210(132–303)	213(135–307)	205(117.5–294.5)	0.237
Pre-TTM Temperature (°C), IQR	36.0(34.9–36.8)	36.2(35.3–36.9), 685 *	35.4(34.2–36.2), 348 *	<0.001
Pre-TTM Shock, n (%)	488(46.1)	283(40.1)	205(58.1)	<0.001
PRTM, n (%)	670(63.3)	444(62.9)	226(64.2)	0.930
Target temperature, n (%)				0.747
32.0-34.0°C	841(79.4)	563(79.7)	278(78.8)	
34.1-36.0°C	218(20.6)	143(20.3)	75(21.2)	
Seizure, n (%)	287(27.1)	202(28.6)	85(24.1)	0.124

Infection, *n* (%)	575(54.3)	390(55.2)	185(52.4)	0.395

Data are presented as the *n* (%) for categorical variables, unless otherwise indicated. IQR, interquartile range; AMI, acute myocardial infarction; TIA, transient ischemic attack; CPR, cardiopulmonary resuscitation; SOFA, sequential organ failure assessment; $PaCO_2$, partial pressure of arterial carbon dioxide tension; HCO_3, arterial bicarbonate; TTM, targeted temperature management; CRP, C-reactive protein; ROSC, return of spontaneous circulation; PRTM, post rewarming temperature management. *, included number for analyses.

Table 2. Multivariate logistic regression analyses of post-rewarming fever and onset-time of post-rewarming fever for mortality at hospital discharge.

	AOR (95% CI)	AOR (95% CI)	AOR (95% CI)
Age, years	1.009 (0.997–1.020)		
Body mass index, kg m^{-2}, IQR	1.044 (1.002–1.088)	1.046 (1.004–1.090)	1.045 (1.003–1.089)
Diabetes mellitus	1.050 (0.690–1.598)		
Angina	0.897 (0.414–1.940)		
Arrhythmia	0.622 (0.251–1.541)		
Renal disease	0.998 (0.514–1.938)		
Pulmonary disease	1.266 (0.648–2.474)		
Cardiac etiology	0.473 (0.321–0.697)	0.488 (0.333–0.715)	0.493 (0.335–0.724)
Downtime, min	1.025 (1.015–1.036)	1.025 (1.015–1.036)	1.085 (1.015–1.160)
Witness	0.752 (0.517–1.094)		
Shockable rhythm	0.535 (0.334–0.857)	0.511 (0.323–0.808)	0.502 (0.316–0.799)
SOFA score	1.114 (1.041–1.191)	1.090 (1.020–1.165)	1.085 (1.015–1.160)
Serum lactate, mg dL^{-1}, IQR	1.005 (0.973–1.039)		
pH	0.221 (0.085–0.569)	0.250 (0.103–0.607)	0.254 (0.104–0.622)
$PaCO_2$, mmHg	0.991 (0.979–1.004)		
HCO_3, mEq dl^{-1}	1.017 (1.006–1.028)		
Peak CRP, mg dL^{-1}, IQR	1.017 (1.006–1.028)	1.017 (1.006–1.028)	1.016 (1.005–1.027)
Pre-TTM Temperature, °C	0.816 (0.728–0.916)	0.826 (0.737–0.926)	0.839 (0.748–0.941)
Pre-TTM Shock	1.671 (1.162–2.403)	1.595 (1.115–2.283)	1.589 (1.107–2.281)
Seizure	0.682 (0.467–0.998)	0.719 (0.494–1.047)	0.720 (0.493–1.050)
PRF	NA	0.732 (0.509–1.052)	NA
No PRF	NA	NA	Reference
PRF within 24 h	NA	NA	0.388 (0.208–0.727)
PRF 24–48 h	NA	NA	0.655 (0.395–1.085)
PRF 48–72 h	NA	NA	1.703 (0.944–3.071)

AOR, adjusted odds ratio; CI, confidence interval; IQR, interquartile range; CPR, cardiopulmonary resuscitation; SOFA, sequential organ failure assessment; $PaCO_2$, partial pressure of arterial carbon dioxide tension; HCO_3, arterial bicarbonate; TTM, targeted temperature management; PRF, post-rewarming fever; CRP, C-reactive protein; ROSC, return of spontaneous circulation; NA, not applicable.

Table 3. Demographics and characteristics of the included patients according to neurologic outcomes at hospital discharge.

	Total (*n* = 1059)	Favorable (*n* = 392)	Unfavorable (*n* = 667)	*p*-Value
Age, years, IQR	57.0 (46.0–68.0)	54 (44–62)	60 (48–71)	<0.001
Male sex, *n* (%)	758 (71.6)	305 (77.8)	453 (67.9)	0.001
Body mass index, kg m^{-2}, IQR	23.2 (20.9–25.7)	23.1 (21.3–25.5), 390 *	23.3 (20.7–25.8), 661 *	0.796
Pre-existing illness				

Hypertension, *n* (%)	367 (34.7)	116 (29.6)	251 (37.6)	0.009
Diabetes mellitus, *n* (%)	238 (22.5)	58 (14.8)	180 (27.0)	<0.001
AMI, *n* (%)	65 (6.1)	28 (7.1)	37 (5.5)	0.353
Angina, *n* (%)	64 (6.0)	36 (9.2)	28 (4.2)	0.001
Congestive heart failure, *n* (%)	37 (3.5)	12 (3.1)	25 (3.7)	0.607
Arrhythmia, *n* (%)	52 (4.9)	20 (5.1)	32 (4.8)	0.883
Renal disease, *n* (%)	80 (7.6)	18 (4.6)	62 (9.3)	0.005
Pulmonary disease, *n* (%)	66 (6.2)	8 (2.0)	58 (8.7)	<0.001
TIA or ischemic stroke, *n* (%)	61 (5.8)	17 (4.3)	44 (6.6)	0.135
Liver cirrhosis, *n* (%)	12 (1.1)	1 (0.3)	11 (1.6)	0.039
Malignancy, *n* (%)	56 (5.3)	20 (5.1)	36 (5.4)	0.888
Cardiac etiology, *n* (%)	662 (62.5)	343 (87.5)	319 (47.8)	<0.001
Downtime, min, IQR	27.0 (16.0–40.0)	18 (13–27)	33 (22–46)	<0.001
Witness, *n* (%)	746 (70.4)	329 (83.9)	417 (62.5)	<0.001
Bystander CPR, *n* (%)	664 (62.7)	266 (67.9)	398 (67.9)	0.008
Shockable rhythm, *n* (%)	388 (36.6)	271 (69.1)	117 (17.5)	<0.001
SOFA score, IQR	11.0 (8.0–12.0)	9 (7–11), 373 *	11 (9–13), 604 *	<0.001
Serum lactate, mg dL^{-1}, IQR	9.4 (5.8–12.5)	9.3 (5.5–12.3), 380 *	9.4 (6.0–12.6), 644 *	0.436
pH, IQR	7.11 (6.94–7.25)	7.22 (7.10–7.31), 373 *	7.04 (6.89–7.18), 650 *	<0.001
$PaCO_2$, mmHg, IQR	47.0 (35.0–69.0)	39.0 (32.0–48.1), 373 *	56.3 (39.0–80.9), 650 *	<0.001
HCO_3, mEq dL^{-1}, IQR	15.3 (12.0–18.8)	15.8 (12.9–19.2), 373 *	15.1 (11.7–18.7), 648 *	0.027
Peak CRP, mg dL^{-1}, IQR	15.8 (9.45–24.45)	13.0 (7.5–19.1), 374 *	17.9 (11.3–27.1), 643 *	<0.001
Time from ROSC to initiation of TTM, min	210.0 (132.0–303.0)	216.5 (146.3–299.0)	208.0 (122.8–304.3)	0.101
Pre-TTM Temperature (°C), IQR	36.0 (34.9–36.8)	36.4 (35.8–37.1), 379 *	35.6 (34.5–36.4), 627 *	<0.001
Peak temperature, (°C), within 72 h. IQR	37.7 (37.2–38.2)	38.0 (37.6–38.3)	37.5 (37.0–38.1)	<0.001
Pre-TTM Shock, *n* (%)	488 (46.1)	136 (34.7)	352 (52.8)	<0.001
PRTM, *n* (%)	670 (63.3)	249 (63.5)	421 (63.2)	0.987
Target temperature, *n* (%)				0.937
32.0-34.0°C	841 (79.4)	312 (79.3)	529 (79.3)	
34.1-36.0°C	218 (20.6)	80 (20.4)	138 (20.7)	
Seizure, *n* (%)	287 (27.1)	49 (12.5)	238 (35.7)	<0.001
Infection, *n* (%)	575 (54.3)	192 (49.0)	383 (57.4)	0.009

Data are presented as the *n* (%) for categorical variables, unless otherwise indicated. IQR, interquartile range; AMI, acute myocardial infarction; TIA, transient ischemic attack; CPR, cardiopulmonary resuscitation; SOFA, sequential organ failure assessment; $PaCO_2$, partial pressure of arterial carbon dioxide tension; HCO_3, arterial bicarbonate; TTM, targeted temperature management; CRP, C-reactive protein; ROSC, return of spontaneous circulation; PRTM, post rewarming temperature management. *, included number for analyses.

Table 4. Multivariate logistic regression analyses of post-rewarming fever and onset-time of post-rewarming fever for unfavorable neurologic outcome at hospital discharge.

	AOR (95% CI)	AOR (95% CI)	AOR (95% CI)	AOR (95% CI)

Age, years	1.031 (1.016–1.047)	1.031 (1.016–1.046)	1.032 (1.017–1.047)	1.032 (1.017–1.047)
Male sex	0.738 (0.468–1.163)			
Hypertension	1.353 (0.822–2.229)			
Diabetes mellitus	0.681 (0.999–2.818)			
Angina	0.416 (0.187–0.923)	0.460 (0.207–1.026)	0.459 (0.205–1.024)	0.439 (0.198–0.974)
Renal disease	0.601 (0.265–1.361)			
Pulmonary disease	0.916 (0.338–2.480)			
TIA or ischemic stroke	0.602 (0.242–1.500)			
Liver cirrhosis	13.864 (0.643–298.891)			
Cardiac etiology	0.274 (0.159–0.471)	0.261 (0.154–0.442)	0.256 (0.151–0.432)	0.261 (0.154–0.442)
Downtime, min	1.055 (1.039–1.070)	1.052 (1.037–1.067)	1.053 (1.038–0.068)	1.052 (1.037–1.067)
Witness	0.638 (0.393–1.036)			
Bystander CPR	1.176 (0.757–1.827)			
Shockable rhythm	0.237 (0.148–0.381)	0.238 (0.149–0.381)	0.230 (0.144–0.366)	0.223 (0.139–0.358)
SOFA score	1.089 (1.008–1.176)	1.105(1.024–1.192)	1.103 (1.023–1.191)	1.100 (1.019–1.188)
pH	0.107 (0.034–0.334)	0.105(1.024–1.192)	0.105 (0.034–0.328)	0.106 (0.034–0.331)
$PaCO_2$, mmHg	1.008 (0.996–1.020)			
HCO_3, mEq dl^{-1}	1.008 (0.942–1.079)			
Peak CRP, mg dL^{-1}, IQR	1.023 (1.009–1.038)	1.022(1.008–1.037)	1.022 (1.008–1.036)	1.021 (1.007–1.036)
Time from ROSC to initiation of TTM, min	1.000 (0.999–1.001)			
Pre-TTM Temperature, °C	0.737 (0.633–0.857)	0.763(0.656–0.888)	0.754 (0.649–0.877)	0.768 (0.660–0.894)
Pre-TTM Shock	0.937 (0.591–1.484)			
Seizure	5.984 (3.655–9.797)	5.939(3.640–9.690)	5.970 (3.661–9.736)	6.024 (3.680–9.861)
Infection	1.573 (1.043–2.372)	1.614(1.070–2.433)	1.546 (1.029–2.321)	1.478 (0.981–2.228)
Peak temperature, °C within 72 h	NA	0.641(0.471–0.872)	NA	NA
PRF	NA	NA	0.623 (0.410–0.945)	NA

No PRF	NA	NA	NA	Reference
PRF within 24 h	NA	NA	NA	0.425 (0.235–0.769)
PRF 24–48 h	NA	NA	NA	0.727 (0.418–1.266)
PRF 48–72 h	NA	NA	NA	0.890 (0.417–1.896)

AOR, adjusted odds ratio; CI, confidence interval; IQR, interquartile range; CPR, cardiopulmonary resuscitation; SOFA, sequential organ failure assessment; $PaCO_2$, partial pressure of arterial carbon dioxide tension; HCO_3, arterial bicarbonate; TTM, targeted temperature management; PRF, post-rewarming fever; CRP, C-reactive protein; ROSC, return of spontaneous circulation; NA, not applicable.
